# Wernicke's Encephalopathy, Wet Beriberi, and Polyneuropathy in a Patient with Folate and Thiamine Deficiency Related to Gastric Phytobezoar

**DOI:** 10.1155/2015/624807

**Published:** 2015-11-30

**Authors:** Nuria Huertas-González, Virgilio Hernando-Requejo, Zaida Luciano-García, Juan Luis Cervera-Rodilla

**Affiliations:** Severo Ochoa Hospital, Leganés, 28911 Madrid, Spain

## Abstract

*Background*. Wernicke's encephalopathy (WE) is an acute neurological disorder resulting from thiamine deficiency. It is mainly related to alcohol abuse but it can be associated with other conditions such as gastrointestinal disorders. This vitamin deficiency can also present with cardiovascular symptoms, called “wet beriberi.” Association with folate deficit worsens the clinical picture.* Subject*. A 70-year-old man with gastric phytobezoar presented with gait instability, dyspnoea, chest pain associated with right heart failure and pericarditis, and folate deficiency. Furosemide was administered and cardiac symptoms improved but he soon developed vertiginous syndrome, nystagmus, diplopia, dysmetria, and sensitive and motor deficit in all four limbs with areflexia.* Results*. A cerebral magnetic resonance imaging (MRI) showed typical findings of WE. He was immediately treated with thiamine. Neurological symptoms improved in a few days and abnormal signals disappeared in a follow-up MRI two weeks later.* Conclusion*. Patients with malabsorption due to gastrointestinal disorders have an increased risk of thiamine deficiency, and folate deficiency can make this vitamin malabsorption worse. An established deficiency mainly shows neurological symptoms, WE, or rarely cardiovascular symptoms, wet beriberi. Early vitamin treatment in symptomatic patients improves prognosis. We recommend administration of prophylactic multivitamins supplements in patients at risk as routine clinical practice.

## 1. Introduction

Wernicke's encephalopathy (WE) is a neurological disorder caused by thiamine (vitamin B1) deficiency. It mainly occurs in alcoholic patients but can also be caused by other circumstances, such as inadequate diet, prolonged parenteral nutrition, vomiting and other gastrointestinal disorders, or increased metabolic needs [1]. Classic clinical presentation is the triad of altered mental status, oculomotor dysfunction, and ataxic gait. However, it can often present differently and may even be associated with cardiovascular symptoms brought on by the same vitamin deficiency, which is known as “wet beriberi” [2].

Folate deficiency can play a role in the development of acute and subacute encephalopathy, either directly [3] or indirectly, by reducing thiamine absorption [4].

We present a case of WE caused by vitamin malabsorption of gastrointestinal origin preceded by wet beriberi and folate deficiency.

## 2. Case Presentation

We report the case of a 70-year-old male patient with a history of hypertension, hyperlipidaemia, peptic ulcer with duodenal stenosis and duodenitis, chronic gastritis, and gastric phytobezoar, “intraluminal mass caused by the accumulation of nondigestible vegetable fibers.”

Three years before the current episode he was admitted to our centre with pericarditis, severe pericardial effusion, bilateral pleural effusion, and atrial fibrillation. While in hospital, he had an episode of confusion and unstable gait which improved spontaneously, although over the following years he was left with mild amnesia-type cognitive impairment and slight gait instability.

The patient attended the emergency room (ER) at our hospital after worsening of his usual degree of gait instability over the last few weeks. Blood tests detected folate deficiency (1.8 ng/mL, with mean corpuscular volume (MCV), 103 fl). This was considered to be the cause of the symptoms and he was discharged with folic acid treatment.

A month later, the patient returned to ER due to persistence of the symptoms. He also reported dyspnoea and chest pain on exertion. He was found to have right heart failure and moderate pericardial effusion and was diagnosed with pericarditis and treated with furosemide, which improved his cardiac symptoms.

Nevertheless, the patient continued to have unstable gait and, a few days later, he developed vertiginous syndrome, bilateral horizontal nystagmus, diplopia without ophthalmoplegia, truncal ataxia, dysmetria, quadriparesis 4/5 in upper limbs and 3/5 in lower limbs, stocking-glove hypoesthesia to touch and pinprick, loss of vibration sense in the lower limbs, and absence of all tendon reflexes.

Lumbar puncture was performed with the following results: leucocytes 2, red cells 9, proteins 44 mg/dL, and glucose 77 mg/dL (plasma glucose 123).

**Figure 1:**
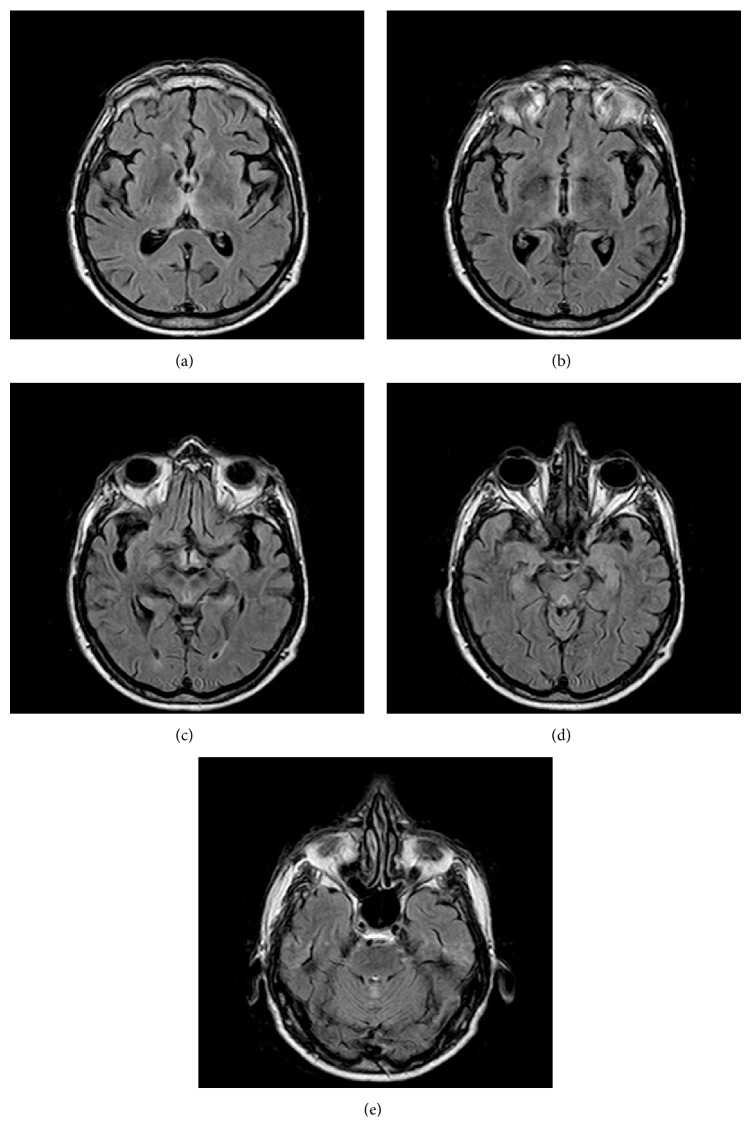
FLAIR axial images of MRI show hyperintensity in (a) the medial region of bilateral thalamus, (b) subependymal grey matter around the third ventricle, (c) third ventricle floor and periaqueductal region, (d) periaqueductal grey matter, and (e) upper cerebellar vermis.

Magnetic resonance imaging (MRI) of the brain was performed showing hyperintensities in T2, FLAIR, and diffusion-weighted sequences in the medial region of both thalamic masses, subependymal grey matter around the third ventricle, periaqueductal grey matter, and cerebellar vermis (Figure 1).

Electromyogram (EMG) showed signs of sensory-motor axonal polyneuropathy.

After other possible causes of the pericarditis had been ruled out and considering the severity of the disease and the possibility that this was a systemic autoimmune process, treatment was started simultaneously with thiamine and immunoglobulins. Within 48 hours the patient improved noticeably and continued to make good progress over the following days.

A brain MRI performed two weeks after the first scan showed that the previously described lesions had disappeared.

At discharge from hospital one month later the patient continued to have minimum distal paresis in all four limbs and stocking-glove paraesthesia without hypoesthesia to touch and pinprick, vibration sense diminished distally in lower limbs, tendon reflexes were weak in upper limbs, both patellar reflexes were very weak, and both Achilles reflexes were absent. He also had mild truncal ataxia but was able to stand up with bilateral support and walked with the aid of a walker with moderate ataxia.

Five months later, he had very slight dysmetria and was getting up and walking independently with a slightly wide-based gait, without cognitive impairment and other focal signs.

## 3. Discussion

Thiamine, or vitamin B1, plays an essential role in normal cell function, growth, and development. Its biologically active form (thiamine pyrophosphate) is a cofactor for many enzymes involved in energy metabolism [5]. Absorption takes place in the duodenum and is enhanced by the presence of folate and magnesium but inhibited by the presence of alcohol [6]. The early phases of deficiency tend to be subclinical or only cause nonspecific symptoms such as headache, fatigue, irritability, or abdominal discomfort [6]; established vitamin deficiency causes primarily neurological and cardiovascular disorders.

Wernicke's encephalopathy is an acute neurological syndrome caused by thiamine deficiency. Classically, it has been described in individuals with chronic alcoholism, but there are other situations that can contribute to vitamin deficiency due to malnutrition [1, 7]: prolonged fasting; anorexia nervosa; prolonged parenteral nutrition; hyperemesis gravidarum; chronic diarrhoea; peptic ulcer; gastrointestinal surgery (especially bariatric surgery); cancer; haemodialysis and peritoneal dialysis.

The classic symptom triad of WE includes confusion, oculomotor dysfunction and ataxic gait. However, only 10% of patients present all three of these symptoms [8] and 19% present none of them. The most common symptom is altered mental state, present in 82% of patients diagnosed at postmortem [9] and encompassing a number of manifestations, from apathy, irritability, inattention and impaired memory and orientation to stupor and coma [10]. Some patients with inadequately treated WE develop a state of permanent amnesia called Korsakoff syndrome [11, 12]. Oculomotor disturbances occur in 29% of cases, the most common finding being nystagmus [9]; bilateral lateral rectus palsy or complete ophthalmoplegia can also occur. Less common is ataxic gait (23%) [9] which may be due to a combination of cerebellar involvement and vestibular dysfunction [10].

Polyneuropathy is found in 11% of patients with WE [10], typically symmetric sensory-motor axonal type affecting the lower limbs and probably contributing to the ataxic gait [13]. Some authors described the neuropathy in patients with folate deficiency alone as an axonal polyneuropathy with predominant involvement in lower limbs but with sensory-dominant pattern and characterized by significantly slower progression than thiamine deficiency neuropathy [14].

In addition to neurological symptoms, thiamine deficiency can also lead to the development of cardiovascular symptoms, which is known as “wet beriberi,” and is characterized by congestive heart failure with reduced peripheral vascular resistance. The diagnostic criteria for this condition [2] include cardiomegaly with normal sinus rhythm, edema, raised venous pressure, nonspecific electrocardiographic ST segment/T wave changes, polyneuropathy [13], absence of any other obvious cause, history of nutritional deficiency, and improvement in the symptoms after the administration of thiamine.

At the same time, in patients with heart failure the use of diuretics such as furosemide is one of the most common treatment options, and it has been shown that administration of this drug can aggravate the thiamine deficiency in situations associated with inadequate vitamin supply due to excessive urinary excretion [15–17].

There are very few published cases in which Wernicke's encephalopathy has been associated with wet beriberi [18–20]. We are reporting the case of a patient with gait instability and mild memory impairment for the past three years, who attended the ER because of worsening of his gait instability and symptoms of congestive heart failure with pericardial effusion. Treating his cardiac symptoms worsened the gait instability and he soon developed additional neurological symptoms such as dizziness, nystagmus, diplopia, dysmetria, and motor deficit in all four limbs, predominantly the lower limbs, with areflexia. This is the first case to be published in which acute Wernicke's encephalopathy is triggered by the use of furosemide to treat heart failure in a patient with a probable prior thiamine deficiency. We believe that, in our patient, the presence of chronic gastritis, gastric phytobezoar, and duodenal stenosis contributed to a chronic thiamine deficiency due to malabsorption. On the other hand, the gastric phytobezoar prevents an adequate folate input due to the lower amount of fiber available, and at the same time duodenitis decreases folate absorption. This demonstrated folate deficiency can further impoverish the absorption of thiamine. The chronic thiamine deficiency was responsible for the slight gait instability that had become worse over the previous months. The persistence of the vitamin deficiency may have also contributed to the development of his cardiac symptoms. We consider it reasonable to assume that the origin of the cardiac and neurological symptoms suffered by the patient three years before was the same as postulated in the most recent admission.

The diagnosis of WE is essentially clinical. However, the difficulty with this is that only a small percentage of patients present with the classic triad and WE is unlikely to be suspected in nonalcoholic patients. Our patient suffered two of the classical symptoms: oculomotor abnormalities and ataxic gait, with a rapidly progressive sensory-motor axonal polyneuropathy suggestive of thiamine deficiency. The fact that symptoms are reversible following administration of thiamine can be diagnostic. In this case, the neurological symptoms improved significantly 48 hours after starting the vitamin therapy.

MRI is considered to be the most valuable method to confirm the diagnosis of WE, as it has a sensitivity of 53% and a specificity of 93% [21]. It typically shows bilateral and symmetrical increased intensity on T2 in the periventricular region of the thalamus, mammillary bodies, floor of the fourth ventricle, and periaqueductal area [22]. There may be atypical lesions in the cerebellum, cranial nerve nuclei, and cerebral cortex, with these most often being found in nonalcoholic patients. The absence of these findings does not rule out the diagnosis. The MRI performed on our patient in the first days of neurological deterioration showed altered signal around the third ventricle and the aqueduct and in cerebellar vermis. These lesions had disappeared by the time of the second MRI several days after starting treatment.

There are no laboratory tests for diagnosing WE. Measuring the erythrocyte thiamine transketolase activity and serum thiamine or thiamine pyrophosphate may contribute to diagnosis [23], but these techniques are not routinely available in most hospitals. We cannot demonstrate a previous thiamine deficiency in our patient because levels were not requested in any of the blood tests prior to starting treatment. However, as mentioned above, the clinical and radiological data and the patient's improvement after the administration of thiamine allowed us to both confirm the diagnosis of thiamine deficiency and rule out a dysimmune origin.

In some cases, a lumbar puncture may be performed to complete the differential diagnosis. Cerebrospinal fluid tends to be normal in patients with WE, although there can be a slight increase in proteins in advanced stages [10, 24]. Our patient had a normal cerebrospinal fluid.

In view of the difficulty for diagnosis, primarily among nonalcoholic patients, and the significant clinical improvement once treatment has been started, early therapeutic action in suspected cases is essential to prevent permanent neurological damage. Under no circumstances should diagnostic tests delay the start of treatment. The most widely accepted recommendations are that patients suspected of having WE should be given 500 mg of intravenous thiamine (dissolved in 100 mL normal saline infused over 30 minutes) three times a day for two consecutive days, followed by 500 mg intravenously or by intramuscular injection once a day for a further 5 days [24]. The patient should then continue on thiamine 100 mg daily, along with magnesium and other vitamins, for several months or until no longer considered at risk. Glucose should not be administered prior to treatment with thiamine in patients with WE, as it can further aggravate this condition [25]. Our patient received treatment early after onset of acute neurological symptoms and has made very good progress.

We consider that in patients with gastric complications and neurological or cardiac symptoms micronutrient status should be assessed and they should early receive multivitamins supplements in combination with symptomatic treatment. We recommend that asymptomatic patients with gastrointestinal disorders receive preventive vitamins supplements as good clinical practice.

## 4. Conclusions

WE is probably an underdiagnosed condition, mainly in nonalcoholic patients. Chronic thiamine deficiency can pass unnoticed clinically, while an established deficiency can manifest in the form of neurological symptoms other than those classically described in WE, or cardiovascular symptoms that we are unlikely to associate with decreased levels of vitamins—wet beriberi. Associated folate deficiency contributes to reducing thiamine absorption. At the same time, administration of furosemide in heart failure can increase deficiency and aggravate the clinical situation. We need to identify patients at risk due to dietary restriction or malabsorption of vitamins, since the early establishment of thiamine treatment rapidly improves acute symptoms and long-term prognosis. Prophylactic administration of vitamin supplements in such risk groups should be introduced as routine clinical practice.
